# Covalent grafting of hyaluronic acid on drug nanocrystals for mucosal delivery

**DOI:** 10.1039/d6tb00506c

**Published:** 2026-06-02

**Authors:** Marta G. Fuster, Octavio E. Fandiño, Akhil Ramesh, Jonnathan A. Coulter, Alejandro J. Paredes

**Affiliations:** a School of Pharmacy, Queen's University Belfast, Medical Biology Centre 97 Lisburn Road BT9 7BL UK a.paredes@qub.ac.uk +44 02890971061

## Abstract

Nanocrystals (NCs) represent an advanced drug delivery platform due to their nearly 100% drug loading capacity, enhanced solubility, and improved tissue penetration. The surface-rich structure of NCs enables chemical modifications, with covalent grafting emerging as a superior strategy to impart stability and introduce targeted functional groups. In this study, we explore the development of curcumin nanocrystals (CUR-NCs) covalently grafted with hyaluronic acid (HA). To enhance mucosal targeting and permeation, HA was chemically grafted to chitosan (CS) on the NCs’ surface *via* EDC/NHS-mediated chemistry. This surface conjugation was confirmed through FTIR and ^1^H-NMR analyses, validating the successful formation of HA–CS–CUR-NCs. The resulting nanoparticles exhibited an average particle size of 110 nm, remaining within the ideal range for mucosal delivery. Importantly, cytotoxicity assays on THP1 monocytes and NIH/3T3 fibroblasts revealed that the HA–CS–CUR-NCs possessed suitable biocompatibility properties. *Ex vivo* mucosal deposition studies using neonatal porcine tissue demonstrated significantly improved mucopenetration with HA-functionalized NCs, achieving 37.3 ± 2.6% drug deposition after 24 hours, compared to only 1.81 ± 1% for non-functionalized CUR-NCs. These findings position HA–CS–CUR-NCs as a promising platform for advanced mucosal drug delivery, combining nanoscale precision with bioresponsive surface chemistry to enhance therapeutic outcomes.

## Abstract 2

Surface-modified curcumin nanocrystals with hyaluronic acid significantly enhance mucosal penetration and drug deposition, offering an advanced targeted delivery platform.

## Graphical abstract

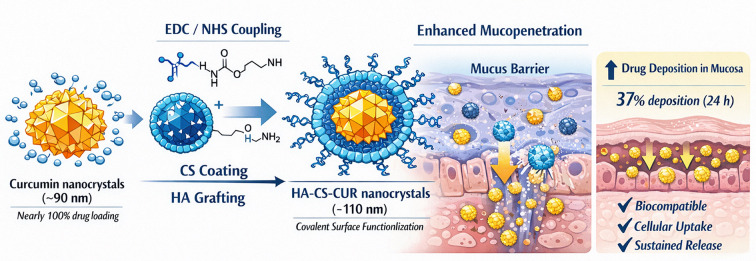

## 1. Introduction

Nanocrystals (NCs) have emerged as a very promising drug delivery platform due to their near 100% drug loading capacity.^1^ NCs are crystalline particles typically ranging in size from 10 to 500 nm, minimally stabilized by surfactants or polymers. Compared to conventional nanoparticles, which often require significant amounts of excipients or carriers, NCs offer improved dissolution rates and higher saturation solubility, making them particularly attractive for high-dose drug delivery with improved pharmacokinetics.^2^ A major advantage of NCs lies in their surface accessibility, which allows for tailored functionalisation strategies. Among these, covalent grafting of functional groups on the NC surface stands out as the preferred method to facilitate targeting, muco-penetration, and controlled release.^3,4^ Additionally, covalent grafting offers superior stability compared to physical adsorption or electrostatic interactions, ensuring durable surface modification under physiological conditions, a crucial aspect for clinical translation.^5–7^ One of the most pressing challenges in drug delivery is overcoming mucosal barriers such as those found in the gastrointestinal, pulmonary, and nasal systems. The dense and viscous nature of mucus restricts the penetration and retention of therapeutic agents.^8^ Efficient mucopenetration is critical for achieving localized drug delivery and enhancing systemic absorption. NCs, owing to their small size and high diffusion rates, exhibit superior muco-penetrative capabilities compared to larger drug carriers.^9,10^ However, this activity can be further enhanced by modifying their surface chemistry.^3,11^ In this context, covalent grafting with hydrophilic and biocompatible polymers such as hyaluronic acid (HA) has been widely explored as a surface modification strategy.^12^ HA is a naturally occurring glycosaminoglycan of the extracellular matrix, characterised by its biocompatibility and hydrophilic nature.^13^ The covalent attachment of HA to NCs’ surfaces can facilitate targeted delivery, while minimizing off-target effects and premature clearance.^14^ HA improves mucosal transport through reduced particle aggregation and enhanced hydrophilic interactions, facilitating diffusion in mucus environments.^15^ This is especially beneficial in nanopharmaceuticals, where uniform distribution and controlled drug delivery are critical for improving therapeutic outcomes and reducing side effects.^16^

To exemplify the potential of this approach, curcumin (CUR) was selected as a model compound. CUR is a natural polyphenolic compound extracted from the rhizome of *Curcuma longa*, and has attracted attention in biomedical research due to its broad spectrum of pharmacological activities.^17^ CUR exhibits potent anti-inflammatory, antioxidant, antimicrobial, and anticancer properties, making it a promising candidate for the treatment of various chronic diseases, including cancer, arthritis, and neurodegenerative disorders.^18^ Despite these benefits, clinical use of CUR remains low due to its poor water solubility (approximately 11 ng mL^−1^ in aqueous solutions), low chemical stability, and rapid systemic elimination. These challenging characteristics ultimately lead to suboptimal bioavailability and limited therapeutic efficacy.^19^ NC formulations have been proposed as an effective strategy to improve the dissolution rate of CUR.^20,21^ Moreover, HA-modified nanoparticles have demonstrated improved drug encapsulation, stability, and controlled release.^12,22^ In addition, the hydrophilic HA coating can reduce particle adhesion and improve mobility within biological fluids. These properties make HA-modified nanoparticles promising for delivering drugs to hard-to-reach tissues, like tumors or inflamed sites, while minimizing systemic side effects.^23^ Ji *et al.*^24^ developed HA-coated CUR-NCs (HA–CUR-NCs) for breast cancer therapy. The HA coating improved the bioavailability of CUR, prolonging the circulation time, and enhanced the uptake in CD44 receptor-overexpressing cancer cells. *In vivo*, NCs exhibited superior anticancer effects with reduced side effects, demonstrating their potential for targeted breast cancer treatment. Zerrillo *et al.*^25^ designed HA-decorated poly(lactide-*co*-glycolide) (PLGA) nanoparticles for osteoarthritis therapy. The HA modification improved binding to CD44 receptors on chondrocytes and enhanced cartilage tissue targeting, while conferring no direct cytotoxicity. Non-invasive imaging confirmed efficient nanoparticle accumulation, highlighting their suitability for targeted osteoarthritis therapies. Seo *et al.*^26^ created multilayered doxorubicin (DOX) and HA cellulose NCs (CNCs) for CD44-targeted delivery. This formulation improved drug penetration, ROS production, and cancer cell death in lung adenocarcinoma models. *In vivo* imaging confirmed enhanced tumor accumulation, demonstrating the potential for targeted chemotherapy. Despite these promising results with HA-functionalised polymeric and lipid-based nanoparticles, a fundamental limitation remains: the relatively low drug loading capacity imposed by the presence of carrier materials, which can reduce the overall therapeutic payload per particle.^27^ In contrast, HA-functionalised NCs uniquely combine the biological advantages of HA-mediated targeting and mucosal interaction with a highly loaded drug core.^28^ This distinction is particularly relevant for poorly soluble drugs requiring high-dose administration, where conventional nanoparticulate systems may suffer from formulation-related limitations.^29^ Moreover, unlike polymeric or lipid matrices that may introduce diffusional barriers, NCs provide a solid drug core that preserves rapid dissolution while HA surface engineering enhances mucus diffusion and receptor-mediated uptake.^30^ Therefore, HA-functionalised NCs represent a structurally distinct and functionally synergistic platform that integrates maximal drug payload, improved mucosal transport, and active targeting within a single carrier-free system.

Given the superior drug loading capability of NCs and the benefits of stable surface functionalisation, this study focused on the covalent grafting of HA onto CUR-NCs. The aim was to systematically synthesize, characterize, and optimize HA-grafted CUR-NCs, evaluating their *in vitro* performance and impact on *ex vivo* mucopenetration in excised porcine mucosa. This work provides a foundation for the development of NC-based drug delivery systems designed to leverage the synergistic advantages of high drug loading, covalent surface engineering, and targeted mucosal delivery.

## 2. Experimental

### 2.1. Materials

All chemicals and materials used in this study were of analytical grade. Curcumin (CUR–CAS RN 458-37-7) was purchased from Tokyo Chemical Industries (London, UK), Poloxamer 188 (POL 188) from BASF chemical company (Ludwigshafen, Germany) and Poloxamer 407 (POL 407). Chemicals such as chitosan (CS–LMW – 50–190 KDa), deacetylated chitin, poly(d-glucosamine) and *N*-hydroxysuccinimide (NHS–CAS 6066-82-6) were purchased from Sigma-Aldrich (Poole, Dorset, UK). 1-Ethyl-3-(3-dimethyllaminopropyl) carbodiimide hydrochloride (EDC–CAS 25952-53-8) was obtained from EMD Millipore Corp (USA) and sodium hyaluronate from Kewpie Corporation (Shibuya-Ku, Tokyo). For the milling process, YTZP Yttria-stabilized zirconia beads of 0.15 and 0.5 mm were purchased from Chemco (Guangfu, China). Parafilm M® was purchased from Bemis Company Inc. (Neenah, USA). Ultrapure water was obtained from a water purification system, Elga Purelab DV 25, Veolia Water Systems (Ireland).

### 2.2. Methods

#### 2.2.1. Optimisation of curcumin nanocrystals

##### 2.2.1.1. Effect of surfactant type

CUR-NCs were prepared by slightly modifying an already published media milling technique^1^ wherein 100 mg of CUR was sandwiched between 4.5 mL of zirconia beads and magnetic bars (25 × 8 mm) in a 10 mL vial. 5 mL of varying strengths (0.5%, 1%, 1.5% and 2% w/v) of P188 and P407 solutions were added. The vials were then completely covered using tin foil and agitated at 1200 rpm for 24 hours. The resultant CUR-NCs were filtered from the milling media using a 300-mesh sieve. 20 µL of the resultant filtrate was diluted with 3 mL of HPLC water in a cuvette and analysed for particle size, PDI and zeta potential.

##### 2.2.1.2. Effect of chitosan concentration

CS, a cationic polysaccharide, was used due to its positive charge, which promotes interactions with negatively charged biological interfaces, and its primary amine groups, which enable covalent coupling with carboxyl-containing molecules such as HA *via* EDC/NHS chemistry. Based on this functional requirement for downstream covalent modification, CS–CUR-NCs were prepared using a media milling technique. Briefly, 100 mg of CUR, 4.5 mL of zirconia beads (0.1–0.2 mm), and two magnetic bars (25 × 8 mm) were added. Varying amounts of POL 188/POL 407 and CS (0.5% w/v solution) were used as summarized in Table 1. The milling was performed at 1200 rpm for 24 h. The resulting NCs were then filtered and characterized as described previously.

**Table 1:** Optimization parameters for chitosan–curcumin nanocrystals: poloxamer and chitosan volumes used

POL 188/POL 407 (% w/v)	Final volume POL 188/POL 407 (mL)	Final volume of 0.5% CS (mL)
0.5	4.75	0.25
1	4.5	0.5
1.5	4	1

#### 2.2.2. Functionalisation of chitosan–curcumin nanocrystals with hyaluronic acid

To functionalize CS–CUR-NCs, HA was activated using a solution of EDC and NHS under stirring at 300 rpm for 5 minutes, then agitated for 60 minutes to form the HA–EDC–NHS intermediate. The molar ratios of both EDC : HA and NHS : HA were approximately 66 : 1, ensuring efficient activation of the HA carboxyl groups. Activated HA was then added dropwise to a suspension of CS–CUR-NCs and stirred at 300 rpm for 16 hours at room temperature. The resulting NCs were collected by centrifugation at 342*g* for 15 minutes, before freeze-drying for 25 hours. The final freeze-dried HA–CS–CUR-NCs pellet was analysed using various physicochemical characterization techniques.

#### 2.2.3. Particle size, polydispersity index and zeta potential

A NanoBrook Omni dynamic light scattering (DLS) analyser (Brookhaven Instruments Corp., Holtsville, NY, USA) was used to determine the particle size, polydispersity index (PDI), and zeta potential of the samples. For the measurements, 20 µL of the prepared NCs were diluted with 3 mL of distilled water in measurement cuvettes. All samples were measured in triplicate, and the results are reported as mean ± standard deviation (SD, *n* = 3).

#### 2.2.4. Characterization

##### 2.2.4.1. Physicochemical characterization

For the physicochemical characterization, samples of CUR-NCs, CS–CUR-NCs, HA–CS–CUR-NCs and PM were analysed using a PerkinElmer Spectrum Two FTIR spectrometer (PerkinElmer Inc., USA). The analysis was carried out in the wavelength range of 4000 cm^−1^ to 500 cm^−1^, averaging 64 scans per spectrum. All major bands obtained were recorded using OMNIC™ Spectra Software (Thermo Fisher Scientific Inc., Waltham, USA). Thermal characterization, including differential scanning calorimetry (DSC) and thermogravimetric analysis (TGA), was carried out using a Q100 DSC instrument (TA Instruments, Bellingham, WA). Scans were conducted from 20 °C to 350 °C at a heating rate of 10 °C min^−1^ under a constant flow of nitrogen (10 mL min^−1^). TGA experiments were carried out between 25 °C and 350 °C at a heating rate of 10 °C min^−1^ and a nitrogen flow of 10 mL min^−1^, using a Q500 instrument (TA Instruments, New Castle, DE, USA). An RM5 Confocal Raman Microscope (Edinburgh Instruments Ltd, Livingston, UK) was used to analyse the Raman spectra of the obtained NCs. Approximately 2 mg of the sample were taken on a glass slide. The grating was set at 300 g mm^−1^, the laser power at 50% and laser wavelength at 785 nm. The spectrum was analysed within the range 0–3200 Raman shift per cm at 5 accumulations each with an exposure time of 1.5 s. Signals were recorded and analysed using the Ramacle® Software (Edinburgh Instruments Ltd, Livingston, UK). Finally, the assessment of ligand attachment was evaluated using proton nuclear magnetic resonance (NMR), ^1^H NMR. Approximately 8 mg of the FD samples were dissolved in 400 µL of DMSO (dimethyl sulfoxide-d6), and then transferred to 7″ NMR sample tubes (Wilmad Economy, NJ, USA). A Bruker Ultrashield 400 spectrometer (Bruker, Leipzig, Germany) was operated at 400 MHz to record the NMR spectra. MestReNova 6.0.2© (Mestrelab Research, Santiago de Compostela, Spain) was used to process the spectra obtained. The observed chemical shifts relative to tetramethylsilane were reported in ppm (*δ*).

##### 2.2.4.2. Microscopy and imaging techniques

Advanced microscopy imaging techniques were employed to characterise CUR-NCs, CS–CUR-NCs and HA–CS–CUR-NCs. NCs were first desiccated to dryness, then deposited onto adhesive carbon tape, and examined using a TM3030 microscope (Hitachi, Krefeld, Germany). A Tabletop TM 3030 scanning electron microscope (SEM) (Hitachi, Tokyo, Japan) was utilised to reveal the topological features of NCs, providing detailed surface morphology data.

#### 2.2.5. HPLC quantification

The detection and quantification of CUR were performed by using HPLC-UV following the protocol reported before.^31^ A ZORBAX Eclipse XDB-C18 column (50 × 4.6 mm internal diameter; 1.8 µm particle size) was selected as the stationary phase. The mobile phase was composed of 80 : 20% (v/v) acetonitrile (ACN) and water (0.1% phosphoric acid). The maxima absorption (*λ*_max_) was fixed at 425 nm. The injection volume was set as 20 µL, and the flow rate was 0.5 mL min^−1^. The linearity of the method was explored between 0.1 and 50 µg mL^−1^ (*r*^2^ = 0.9999). The limits of detection and quantification were 0.27 and 0.81 µg mL^−1^, respectively. HPLC analysis was applied for two distinct purposes in this study: (i) determination of CUR content in the formulated nanosuspensions (drug assay) and (ii) quantification of CUR in experimental samples, including *in vitro* release and *ex vivo* mucosal deposition studies.

##### 2.2.5.1. Determination of curcumin content in the formulated nanosuspensions

To determine the CUR content in the obtained formulations, 20 µL of the resulting nanosuspension was dissolved in 980 µL of ACN. The resulting solution was centrifuged at 16 602*g* for 15 minutes and subsequently filtered using a 0.22 µm filter (Millipore, USA) for subsequent quantification by high-performance liquid chromatography (HPLC).

#### 2.2.6. *In vitro* release study

A dialysis membrane technique was used to evaluate *in vitro* release profiles comparing pure CUR drug, a physical mixture (PM) of each nanoformulation, and the NCs. The release medium was a phosphate buffer at pH 6.4 (simulating saliva), supplemented with 2% w/v Tween 80 and 0.5% ascorbic acid to prevent degradation of the drug.^32^ After confirming sink conditions, samples containing approximately 3 mg of CUR were added to preactivated (PBS for 1 h) dialysis membrane bags (molecular weight cut-off: 12 000–14 000 Da; Spectra-Por, Spectrum Medical Industries, Los Angeles, CA, USA). The bags were sealed with plastic clips and incubated in an ISF 7100 orbital shaker (Jeio Tech, MA, USA) at 37 °C and 100 rpm. At predetermined time intervals (0 h, 1 h, 3 h, 6 h, 9 h, 1 day, 2 days, 3 days, 7 days, 14 days and 28 days), 1 mL of release media was withdrawn and replaced with fresh buffer. CUR content was quantified using HPLC (section 2.2.5). All experiments were conducted in triplicate, with results expressed as mean ± standard deviation (*n* = 3).

#### 2.2.7. Cell culture experiments

THP1 human monocytic and NIH/3T3 cell line fibroblast cell line from a mouse were purchased from the American type culture collection (ATCC, Manassas, VA, USA) and stored according to the supplier's guidelines. THP1 cells were kept in Roswell park memorial institute medium (RPMI) and NIH/3T3 in Dulbecco's modified Eagle medium (without phenol red) supplemented with 10% (v/v) heat-inactivated fetal bovine serum. Cells were maintained in T75 culture flasks, in a humidified environment with 5% CO_2_/95% air at 37 °C. On reaching 80% confluences, the cells were detached using 0.25% trypsin-EDTA, then either seeded for experiments or passaged to maintain stocks.

##### 2.2.7.1. Viability assay

The cytotoxic effects of CUR-NCs, CS–CUR-NCs, and HA–CS–CUR-NCs on NIH/3T3 cells were tested using the Alamar Blue® assay (Thermo Fisher Scientific, Waltham, MA, USA). Cells were seeded in 96-well plates at a concentration of 5 × 10^4^ cells per well. Twenty-four hours post seeding, cells were treated with fresh medium (negative control) or various concentrations of NC-containing media for 24 h. Post treatment, excess NCs were replaced with complete media containing 10% Alamar blue solution for 4 h. Fluorescence levels, positivity correlating with viable cell number, were measured using a FLUOstar Omega (BMG LABTECH GmbH, Freiburg, Germany) microplate spectrophotometer at an excitation wavelength of 530 nm and an emission wavelength of 590 nm. Data points were collected over at least three independent experiments.

##### 2.2.7.2. Analysis of cellular uptake of nanocrystals by flow cytometry

1 × 10^4^ cells were seeded into individual wells on a 6-well plate, and then incubated at 37 °C for 24 h to allow attachment. The culture medium was replaced by a fresh medium containing NCs at a concentration equivalent to 120 µg mL^−1^ of CUR and incubated for 24 h. This concentration was selected based on preliminary cytotoxicity studies to ensure a non-toxic yet pharmacologically relevant dose for subsequent biological evaluation. Following incubation, NCs were removed by washing in PBS (×3), cells were detached using 0.25% trypsin-EDTA, and then fixed in 4% paraformaldehyde for 20 min. Cellular uptake was assessed by quantifying the associated fluorescence using a Becton–Dickinson FACSCalibur flow cytometer (New Jersey, United States). Cell-associated fluorescence was quantified using a Becton–Dickinson FACSCalibur flow cytometer (New Jersey, United States). The intrinsic fluorescence of CUR was used to monitor cellular uptake.^33^ Untreated cells were used as controls to determine background autofluorescence.

#### 2.2.8. *Ex vivo* experiments

##### 2.2.8.1. Mucosal deposition

As detailed below, the *ex vivo* mucosal deposition studies of NCs were performed in excised buccal neonatal porcine mucosa. The mucosa was cleaned with pH 7.4 buffer and gently cut using a sterile scalpel. Then, following a previously published methodology, each mucosa section was placed in a weigh boat containing tissue paper soaked in buffer.^34^ 3D printed resin rings with an inner diameter of 6 mm and a height of 5 mm were attached to the mucosa with glue using manual force for 30 s. Inside the ring, 30 µL of CUR-NCs, CS–CUR-NCs and HA–CS–CUR-NCs were added. The system was then sealed with Parafilm M® to prevent evaporation and then placed in an incubator at 37 °C. After 24 h, the rings were removed, the mucosa was rinsed with 1 mL of pH 7.4 mucus buffer, and the excess formulation was wiped with a clean tissue paper wetted with mucus buffer. Subsequently, a 6-mm diameter punch biopsy was used to obtain a circular section of mucosa from which the drug was extracted. This experiment was carried out in triplicate. Each circular section of mucosa was placed in a 2-mL Eppendorf tube with two stainless steel beads (0.5 cm diameter, Qiagen, Hilden, Germany) and 500 µL of purified water. Tubes were then placed in a TissueLyser® LT (Qiagen, Hilden, Germany) and processed for 10 min at 50 Hz to hydrate the mucosa. Next, 1 mL of acetonitrile was added to each tube, and the process was repeated for another 10 min at 50 Hz to extract the drug. The homogenized tissue was then centrifuged at 14 462*g* for 10 min (Sigma microtube centrifuge SciQuip Ltd, Shropshire, UK), and the CUR content in the supernatant was quantified by HPLC using the method detailed in Section 2.14. Skin without treatment was used as a control.

##### 2.2.8.2. Fluorescence microscopy

Specimens of the mucosal tissues obtained from the drug deposition experiment in porcine mucosa were imaged using a multiphoton microscope (MPM) (Leica TCS SP8 multi-photon excited fluorescence upright microscope, Leica Microsystems Ltd, Milton Keynes, UK) to observe the distribution of CUR in the scleral tissue. Different NC formulations were evaluated, in addition to the physical mixture of HA–CS–CUR-NCs. Samples were analysed after 2 h, 4 h, and 24 h of exposure to assess the penetration behaviour and time-dependent distribution of NCs within the tissue.

#### 2.2.9. Statistical analysis

Statistical analysis was performed using GraphPad Prism© software (version 8.0, GraphPad Software Inc., San Diego, California, USA). One-way ANOVA followed by Tukey's *post hoc* test was performed to compare the particle size and surface charge among CUR, CUR–CS, and CUR–CS–HA NC formulations.

## 3. Results and discussion

### 3.1. Optimisation of nanocrystals

#### 3.1.1. Stabilizer selection study

**Fig. 1:**
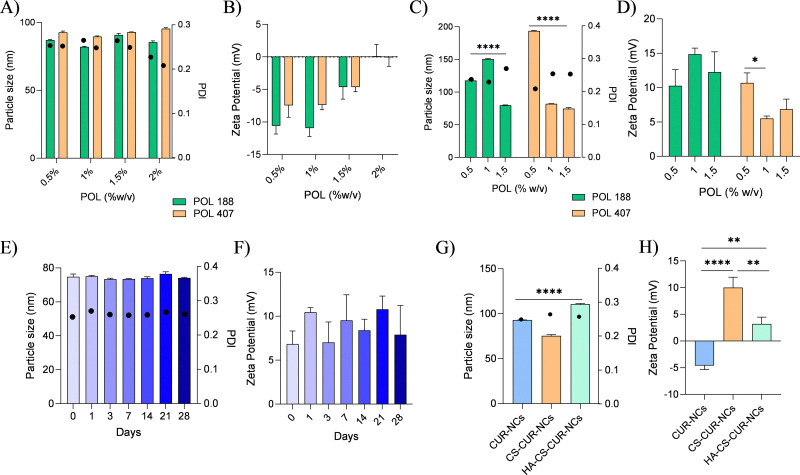
Particle size (represented as bars) and polydispersity index (represented as circles) determined by dynamic light scattering (intensity distribution), along with zeta potential measurements for different nanocrystal formulations. Panels (A) and (B) correspond to stabiliser selection studies; (C) and (D) to chitosan optimisation studies; (E) and (F) to stability studies; and (G) and (H) to functionalised nanocrystals. Data are presented as mean ± standard deviation (SD) from three independent experiments. Statistical analysis was performed using one-way ANOVA followed by Tukey's *post hoc* test. Statistical significance is indicated as follows: *p < 0.05, **p < 0.01, ****p < 0.0001.

The concentration and choice of stabilizer used to synthesize CUR-NCs by media milling were determined by comparing their particle size, polydispersity index (PDI) and zeta potential. Both POL 188 and POL 407 were tested at four concentrations (0.5% w/v, 1% w/v, 1.5% w/v and 2% w/v). The results obtained regarding particle size showed no significant differences across any of the studied POL concentrations, nor when comparing the two types of poloxamer used (POL 188 and POL 407). For example, at a concentration of 1.5% w/v, both POL 188 and POL 407 displayed comparable particle sizes (90.81 ± 1.0 nm and 92.94 ± 0.09 nm, respectively) and similar PDI values (0.264 and 0.249, respectively). In contrast, at the highest concentration (2% w/v), particle sizes (85.43 ± 1.01 nm for POL 188 and 95.42 ± 0.68 nm for POL 407) and zeta potential values (0.13 ± 1.78 mV for POL 188 and −0.09 ± 1.36 mV for POL 407) were not altered, as shown in Fig. 1A. In terms of zeta potential (Fig. 1B), a distinct trend was observed; as the concentration of POL increased, the absolute value of the zeta potential became less negatively charged regardless of the type of POL used. This indicates that concentration, rather than the specific type of POL, influenced the reduction in zeta potential. This effect may be attributed to the non-ionic nature of these polymers, which form a steric stabilisation layer on the surface of NCs. At higher concentrations, increased surface coverage leads to partial shielding of surface charges, resulting in a shift of the measured zeta potential towards neutral values.^35^ For instance, the zeta potential shifted from −10.94 ± 1.28 mV (0.5% POL 188) and −7.44 ± 1.84 mV (0.5% POL 407) to near-neutral values at the 2% concentration (0.13 ± 1.78 mV and −0.09 ± 1.36 mV, respectively). Such a substantial reduction in zeta potential values, approaching neutrality, suggests particle instability and the potential for aggregation. This aligns with the literature findings, which report that nanoparticles with zeta potential values near zero are prone to agglomeration due to insufficient electrostatic repulsion.^36^ As the zeta potential moves closer to neutral, electrostatic stabilisation diminishes, leading to destabilisation of the particle suspension. Considering this, particles synthesised with the highest concentration of POL were deemed unsuitable for further investigation. Despite a relatively stable PDI (0.227 for POL 188 and 0.208 POL 407), the observed particle size and significantly reduced zeta potential render these formulations unfit as stabilisers.

#### 3.1.2. Chitosan-coated curcumin nanocrystals

Based on the results obtained from the stabiliser selection study (3.1.1), three concentrations (0.5%, 1% and 1.5% w/v) of both POL 188 and POL 407 were chosen to prepare six different CUR-NCs with a 0.5% w/v CS solution. From Fig. 1C and D, it can be observed that the particle size of the NCs decreases significantly (*p* < 0.0001) with the addition of CS as the percentage of POL increases. For instance, in the case of POL 188, the particle size reduces from 117.06 ± 1.27 nm to 79.79 ± 0.46 nm. A similar trend is observed for POL 407, showing a decrease from 193.00 ± 0.72 nm at lower POL concentrations to an optimal size of 74.85 ± 1.53 nm at 1.5% w/v. While both formulations demonstrated promising results for further experimentation, POL 407 was ultimately selected due to its superior final characteristics, including a lower PDI of 0.261 and a positive zeta potential of 9.53 ± 2.93 mV. The 1.5% w/v concentration of POL 407 was selected as the optimal formulation based on the overall balance of physicochemical properties, including the smallest particle size, a low PDI, and a favourable positive zeta potential, which together indicated improved colloidal stability compared to the other tested concentrations.

##### 3.1.2.1. Stability study data for chitosan–curcumin nanocrystals

Stability studies for the optimised CS–CUR-NCs were conducted at 4 °C over a 28-day period. The particle size results remained consistently below 80 nm across the full study period. Similarly, PDI values (Fig. 1E dots) remained below 0.3, indicating a stable size distribution. Zeta potential measurements (Fig. 1F) displayed minor fluctuations over time but consistently remained positive.

### 3.2. Functionalised nanocrystals with hyaluronic acid

#### 3.2.1. Dynamic light scattering

The data obtained from the particle size and zeta potential analyses are presented in Fig. 1G and F. Comparing across samples, it was evident that CS was successfully conjugated forming the CS–CUR-NCs sample, as the zeta potential following CS conjugation switched from negative (−4.62 ± 0.74 mV) to positive (10.02 ± 1.93 mV). This shift is consistent with the presence of numerous amino groups along the CS backbone, which confer a positive surface charge and facilitate subsequent derivatization. A slight decrease in the particle size from 92.94 ± 0.09 nm to 75.47 ± 1.19 nm was also observed. These changes in NCs' physicochemical properties following surface conjugation are attributed to electrostatic interactions imparted by CS, increasing repulsive forces.^37^ Importantly, PDI values also remained consistently below 0.3 for the three samples.

#### 3.2.2. Attenuated total reflectance-Fourier transform infrared spectroscopy

**Fig. 2:**
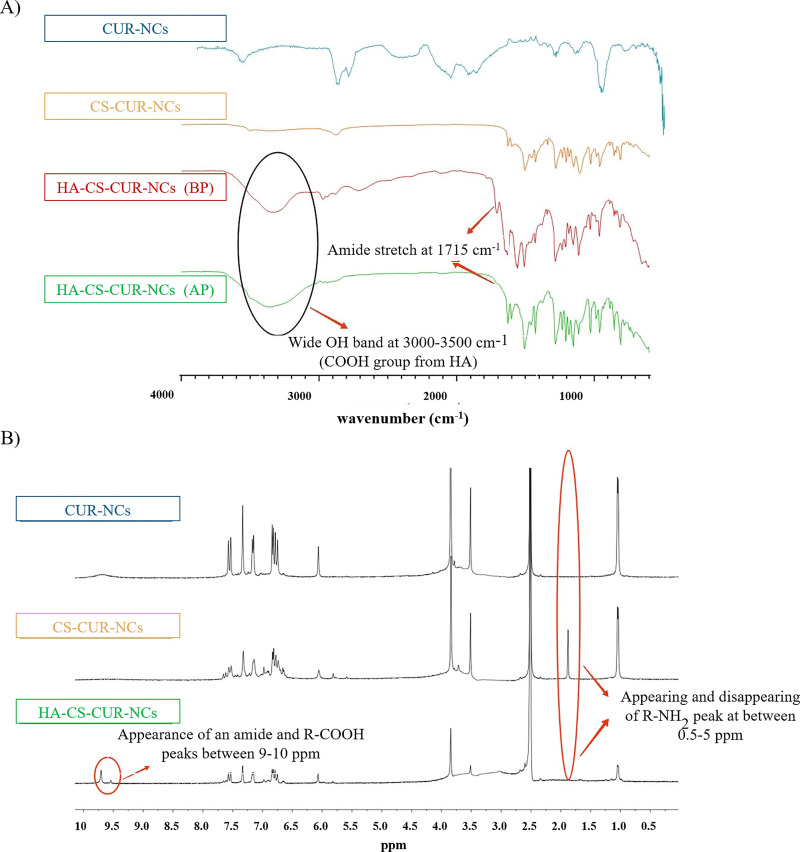
ATR-FTIR spectra (A) and ^1^H-NMR spectra (B) of CUR-NCs, CS–CUR-NCs, and HA–CS–CUR-NCs. The spectra are presented to confirm the chemical composition and successful surface modification of the nanocrystals. In the FTIR spectra, BP refers to “before purification” and AP to “after purification”. Characteristic peaks corresponding to the different components and functional groups are highlighted and assigned.

ATR-FTIR spectroscopy was performed to confirm the successful coating of HA onto the surface of CS–CUR-NCs. The spectra obtained (Fig. 2) revealed key characteristic bands for each analysed sample. A broad band between 3000 cm^−1^ and 3500 cm^−1^ indicated the presence of the –COOH group, suggesting the successful incorporation of HA. Additionally, distinct peaks were identified for CS (amine stretching at 3355 cm^−1^ and amide I stretch at 1652 cm^−1^), POL 407 (polyethylene oxide band at 1100 cm^−1^ and polypropylene stretch at 2897 cm^−1^), and CUR (OH stretch at 3525 cm^−1^, C=C at 1603 cm^−1^, and C=O at 1650 cm^−1^). A comparison between the sample before and after purification (Fig. 2A) highlighted the presence of an amide band at 1715 cm^−1^, indicating a successful EDC–NHS coupling reaction. This band is associated with the formation of a urea byproduct, which serves as further confirmation of the functionalization process. After purification, all major characteristic peaks were still present; however, the intensity of the amide band at 1715 cm^−1^ was notably reduced, suggesting that a significant portion of the urea byproduct had been removed.^38^ While theoretically, a strong amide band (between 1650 cm^−1^ and 1750 cm^−1^) would still be expected even after purification, the presence of a small shoulder band at 1715 cm^−1^ suggests that HA remains attached to CS–CUR-NCs. The observed reduction in absorbance intensity may be attributed to factors affecting the efficiency of the EDC–NHS coupling reaction, including variations in pH, lower reagent concentrations, or temperature fluctuations during synthesis. These parameters could influence both the reaction yield and the intensity of the bands observed in the spectra.^37^ Taken together, these results suggest the successful formation of an amide bond, indicating that covalent coupling successfully occurred. Nevertheless, further characterization techniques, including ^1^H-NMR analysis, were used to confirm the structural modification, ensuring the reliability of the findings.

#### 3.2.3. Proton nuclear magnetic resonance analysis

^1^H-NMR analysis was conducted to complement the DLS and ATR-FTIR data, to gain a deeper understanding of the molecular interactions and structural integrity of the NCs after surface modification. Three samples CUR-NCs, CS–CUR-NCs, and HA–CS–CUR-NCs were analysed, revealing various proton chemical shifts, as shown in Fig. 2B. A strong peak at 2.5 ppm was observed in all spectra, corresponding to DMSO used in sample preparation. In the CS–CUR-NCs spectrum, an upfield peak between 0.5 and 2.5 ppm was detected, suggesting the presence of an R–NH_2_ functional group from CS.^39^ However, this peak was absent in the spectrum of HA–CS–CUR-NCs (Fig. 2B), indicating that the amine groups had reacted, confirming the functionalization of CS with HA. This conclusion was further supported by the appearance of two distinct peaks between 9.0 and 10.0 ppm in the HA–CS–CUR-NCs spectrum, which, based on the literature, correspond to the R–COOH and R–CO–NHR groups.^40^ These shifts provide strong evidence of the successful attachment of HA onto CS–CUR-NCs *via* the EDC–NHS coupling reaction. Additionally, all three samples exhibited peaks between 6.0 and 8.0 ppm, corresponding to the aromatic protons of CUR,^41^ indicating that the core nanocrystal structure remained intact throughout the functionalization process. The overall spectral changes confirm the successful conjugation of HA onto CS–CUR-NCs and validate the efficiency of the EDC–NHS coupling chemistry.

#### 3.2.4. Thermal analysis

**Fig. 3:**
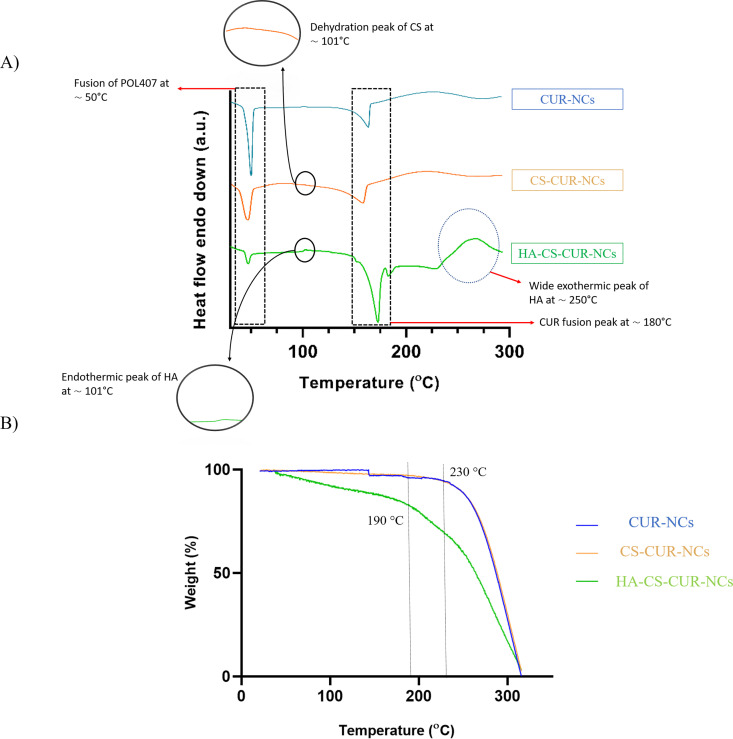
Thermal characterisation of CUR-NCs, CS–CUR-NCs, and HA–CS–CUR-NCs. Panel (A) shows differential scanning calorimetry (DSC) thermograms, while panel (B) presents thermogravimetric analysis (TGA) profiles over the studied temperature range. These analyses were used to evaluate thermal stability and potential interactions between components. Characteristic thermal transitions and degradation events are indicated.

DSC thermograms were recorded for CUR-NCs, CS–CUR-NCs and HA–CS–CUR-NCs. The results (Fig. 3) displayed fusion peaks for POL 407 (∼50 °C) and CUR (∼180 °C) across all samples, confirming their presence. In CS–CUR-NCs (Fig. 3A), a broad peak around 101 °C was observed, indicative of loss of water.^42^ The thermogram for HA–CS–CUR-NCs showed two additional peaks, one endothermic and one exothermic, alongside the fusion peaks common to all samples. The broad endothermic peak at ∼97 °C and the exothermic peak at ∼250 °C suggest the presence of HA. However, since CS also exhibits an endothermic peak near 100 °C, the possibility of peak overlap between CS and HA was considered. This risk is mitigated by the presence of an exothermic peak at ∼250 °C, a feature unique to HA, further supporting successful incorporation.

TGA of CUR-NCs, CS–CUR-NCs and HA–CS–CUR-NCs was performed to assess their thermal behaviour. As shown in Fig. 3B, the thermograms of CUR and CS-NC formulations exhibited a relatively stable mass up to approximately 230 °C, after which an initial mass loss of approximately 8% was observed, indicating the onset of decomposition. This behaviour is typical for the individual components, with the weight loss likely attributed to the degradation of CUR and CS.^43^ In contrast, the HA–CS–CUR-NCs formulation showed a more pronounced and earlier onset of mass loss, beginning around 190 °C. This suggests that HA in the formulation undergoes decomposition at a lower temperature compared to CS and CUR, which is consistent with the known thermal behaviour of HA.^44^ The faster degradation observed for HA–CS–CUR-NCs could be due to the specific characteristics of HA, which can be more thermally labile than CS. Despite this, all formulations remained thermally stable even above their respective drug melting temperatures, indicating that the NC formation process *via* media milling did not affect the thermal stability of the active ingredients. These results suggest that the incorporation of HA does not significantly compromise the overall thermal stability of the formulation but may influence its decomposition dynamics.

#### 3.2.5. Microscopy images

**Fig. 4:**
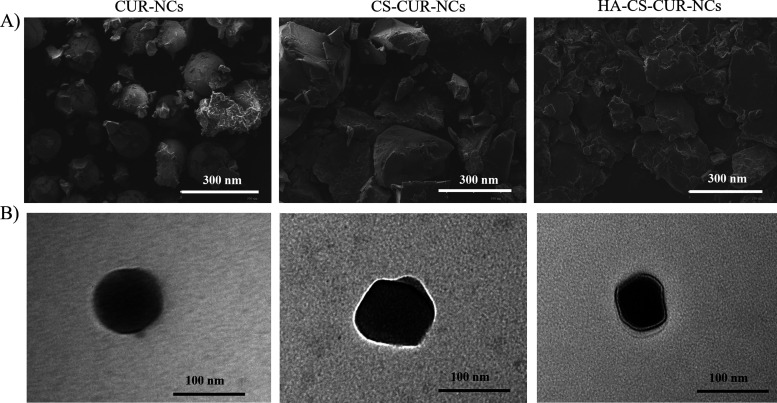
Morphological characterisation of nanocrystals. Panel (A) shows scanning electron microscopy images (scale bar: 300 µm), and panel (B) shows transmission electron microscopy images (scale bar: 100 µm) of CUR-NCs, CS–CUR-NCs, and HA–CS–CUR-NCs. These images illustrate particle shape, size, and surface morphology.

SEM and TEM were used to characterise the morphology of CUR-NCs, CS–CUR-NCs and HA–CS–CUR-NCs. SEM analysis (Fig. 4A) of CUR-NCs revealed spherical structures, likely due to the use of POL as a surfactant.^45^ In the case of CS–CUR-NCs, SEM images exhibited platelet-like formations, potentially attributable to CS deposition, despite the continued presence of POL as a stabiliser. This is consistent with prior studies reporting morphological alterations in nanoparticles following CS coating, where polymeric layers can influence surface roughness and structure.^46^ Upon chemical modification with HA, the observed platelets appeared larger, which could be explained by the centrifugation step necessary for the conjugation reaction. This process may lead to the partial removal of POL, thereby exposing or restructuring the CS. However, it is important to note that these visible structures do not directly represent the NCs themselves. During sample drying, aggregation of the stabiliser occurs, forming a matrix in which the NCs are embedded. Due to the limited resolution of SEM, typically at the micrometre scale, it is extremely difficult to visualise individual NCs within this matrix. For this reason, TEM was employed to gain more detailed insight into the nanostructures (Fig. 4B). CUR-NCs were observed to be spherical. In CS–CUR-NCs samples, phase contrast TEM revealed a distinguishable outer layer, suggestive of CS adhesion *via* electrostatic interactions. Finally, the TEM analysis of HA–CS–CUR-NCs demonstrated the presence of a well-defined additional layer surrounding the NCs, indicative of successful conjugation with HA. These observations confirm that the chemical modification was effective and that TEM is a valuable tool for visualising the added HA layer, providing direct evidence of the surface functionalisation achieved in this study.

#### 3.2.6. *In vitro* cell culture assays

**Fig. 5:**
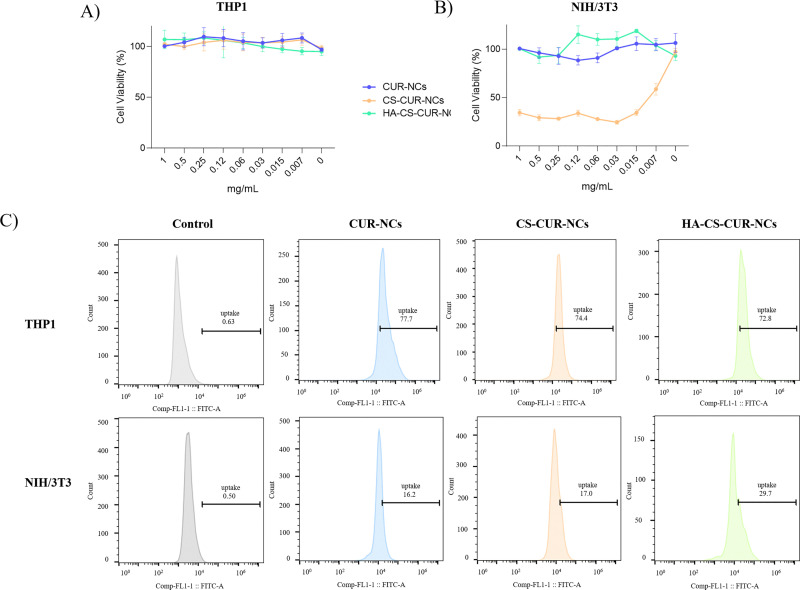
Cytotoxicity evaluation of nanocrystals. Panels (A) and (B) show the effect on THP-1 and NIH/3T3 cell viability, respectively, following treatment with the different formulations. Panel (C) presents the quantitative analysis obtained by flow cytometry for both cell lines. Data are expressed as mean ± standard deviation (SD) from three independent experiments.

Cell viability was evaluated using the Alamar Blue assay in THP1 monocytes and NIH/3T3 fibroblasts, testing CUR-NCs, CS–CUR-NCs, and HA–CS–CUR-NCs. While these *in vitro* models do not represent the mucosal epithelium directly, they provide valuable insights into cytocompatibility, immune cell interaction, and general NC behaviour at the cellular level. THP1 monocytes are relevant due to the presence of innate immune cells within mucosal tissues, which play a role in NC clearance, inflammation, and antigen sampling. NIH/3T3 fibroblasts, although not directly involved in NC uptake at the mucosal interface, reside in the underlying connective tissue and are crucial for evaluating potential subepithelial toxicity and inflammatory signalling.^47^ These two models thus complement the mucopenetration studies by providing mechanistic understanding of how surface modifications affect cell–NCs interactions. The results showed in Fig. 5A demonstrate that for THP1 cells, none of the NCs tested exhibited direct cytotoxic effects, with viability remaining at ∼100%. This finding suggests CUR-NCs would not negatively impact circulating monocytes, immune cells that often form the first line of defence against pathogens. Similarly, CUR-NCs and HA surface-modified CUR-NCs exhibited no cytotoxicity in NIH/3T3 fibroblasts (Fig. 5B). However, CS-coated NCs without HA modification (CS–CUR-NCs) exhibited a slight increase in sensitivity, with a trend towards a dose-dependent cytotoxic response. This behaviour has been previously reported in similar studies, where HA surface modification was shown to improve bioavailability and biocompatibility.^48^ To further investigate the interaction between NCs and relevant cell types, cellular uptake was assessed 24 h post-treatment using flow cytometry (Fig. 5C). In THP1 cells, uptake was uniformly high compared to the control for all NCs tested: 77.7% for CUR-NCs, 74.4% for CS–CUR-NCs, and 72.8% for HA–CS–CUR-NCs, suggesting that these cells internalise NCs effectively regardless of surface modification. This behaviour is consistent with the high phagocytic capacity of THP1 cells^49^ and confirms that surface engineering does not impair internalisation in professional phagocytes. In contrast, NIH/3T3 fibroblasts exhibited a surface modification-dependent uptake profile: internalisation increased by 16.2% for CUR-NCs, 17% for CS–CUR-NCs, and 29.7% for HA–CS–CUR-NCs relative to control. The enhanced uptake observed for HA–CS–CUR-NCs may be attributed to receptor-mediated endocytosis *via* CD44, which is overexpressed in many cell types and is known to mediate HA binding and internalisation.^50,51^ The non-cytotoxic nature of these NCs ensures that their internalization does not adversely affect the viability, supporting their suitability for biomedical applications.

#### 3.2.7. *In vitro* release profiles and mucosal deposition

**Fig. 6:**
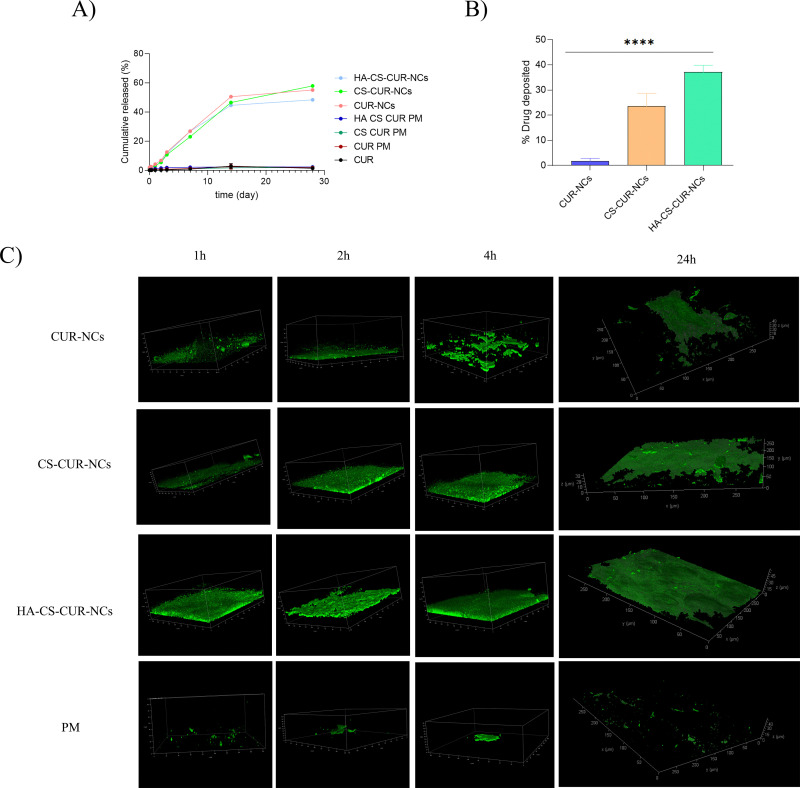
*In vitro* release and mucosal interaction studies. Panel (A) shows the *in vitro* release profiles of curcumin from pure drug, physical mixture, and nanocrystal formulations using a dialysis membrane method (MWCO 12 000–14 000 Da) in phosphate buffer (pH 6.4) supplemented with 2% w/v Tween 80 and 0.5% ascorbic acid, at 37 °C and 100 rpm. Samples containing 3 mg of curcumin were used. Panel (B) presents the mucosal deposition assay performed on excised neonatal porcine buccal mucosa after 24 h of incubation at 37 °C. A volume of 30 µL of each formulation was applied within 3D-printed rings (6 mm diameter), and drug deposition was quantified by HPLC after tissue extraction. Data are expressed as mean ± standard deviation (SD) from three independent experiments. Statistical analysis was performed using one-way ANOVA followed by Tukey's *post hoc* test, with significance indicated as ****p < 0.0001. Panel (C) displays representative transmitted light images obtained by multiphoton microscopy of porcine mucosa treated with CUR-NCs, CS–CUR-NCs, HA–CS–CUR-NCs, and PM at different time points (2, 4, and 24 h) at 37 °C, illustrating their interaction and penetration within the tissue.

The *in vitro* release profiles of CUR, PMs, and NCs are shown in Fig. 6A. As expected, pure CUR exhibited a slow dissolution rate, with only 2.92 ± 1.74% of the drug released by the end of the study. The physical mixtures showed no significant improvement over the pure drug, with CUR PM, CS PM, and HA PM reaching 2.49 ± 1.45%, 1.96 ± 1.07%, and 2.29 ± 1.66% drug release, respectively. These results indicate that the presence of the compounds alone did not significantly enhance the dissolution rate of CUR. In contrast, the CUR-NCs demonstrated a markedly improved dissolution profile, with approximately 55.11 ± 5.20% of the drug being released after 28 days. Similarly, CS–CUR-NCs reached 57.89 ± 5.82%, while HA–CS–CUR-NCs achieved 48.35 ± 6.02% release. These findings highlight two key points: first, that the PMs serve as appropriate controls, confirming that the observed enhancement in drug release is attributable to the NC formulation rather than to the individual components alone; and second, that the nanoformulations are both effective and efficient in increasing the dissolution rate. The increased dissolution rate and saturation solubility observed with NCs can be attributed to an enlarged surface area, which plays a critical role in dissolution kinetics, described by the Noyes–Whitney equation.^52^ Moreover, the sustained drug release profile is a promising characteristic of these systems. It is hypothesized that, despite natural clearance mechanisms at the mucosal level, NCs may remain accumulated in the mucosa, contributing to prolonged drug availability.

#### 3.2.8. *Ex vivo* mucosal deposition and fluorescence assay

The results of the mucosal deposition assay on neonatal porcine mucosa were obtained as follows. Patches were cut into 5 mm diameter discs, with the drug content measured for each formulation. The drug content for CUR-NCs was 18.01 ± 0.11 mg mL^−1^, for CS–CUR-NCs it was 18.54 ± 1.41 mg mL^−1^, and for HA–CS–CUR-NCs it was 2.21 ± 1.14 mg mL^−1^. After 24 hours of contact between the mucosa and the nanosuspension, the drug deposition was quantified. The results showed that 1.81 ± 1% of the drug from CUR-NCs was deposited in the mucosa, while 23.7 ± 5% of the drug from CS–CUR-NCs and 37.3 ± 2.6% from HA–CS–CUR-NCs were deposited, with the latter showing a significantly higher deposition compared to the CUR-NCs formulation (Fig. 6B). The poor penetration of unmodified CUR-NCs may result from their limited ability to interact with the mucus components or from rapid clearance by the mucus layer. Moreover, in immune-cell-rich environments such as mucosal tissues, it is plausible that a fraction of nanoparticles may be internalised by resident macrophages, thereby reducing the number of particles reaching deeper tissue layers. This hypothesis is supported by previous studies demonstrating that macrophages contribute significantly to nanoparticle sequestration and clearance within mucosal barriers.^53^ In contrast, the enhanced deposition observed with the CS–CUR-NCs and HA–CS–CUR-NCs formulations can be attributed to the incorporation of CS and HA, which are known to improve the permeation and adhesion of nanoparticles to mucosal surfaces. CS, a natural polymer with a positive charge, enhances the interaction with the negatively charged cell membranes, facilitating the penetration of drug-loaded nanoparticles through the mucosa.^54^ Additionally, HA, which exhibits mucoadhesive properties, can increase the residence time of NCs at the mucosal surface, promoting higher drug absorption. These findings are consistent with the literature on nanoparticle-based drug delivery systems, where smaller particle sizes enhanced mucoadhesion, leading to improved drug permeation across biological membranes.^55,56^ Therefore, the improved deposition achieved with HA–CS–CUR-NCs reflects not only enhanced mucoadhesion, but also an ability to circumvent immune clearance mechanisms to a certain extent, highlighting their potential for mucosal drug delivery applications. A fluorescence study was conducted using neonatal porcine buccal mucosa maintained at 37 °C after application of the same dose previously employed in the release experiments. Tissue sections were collected and analysed by multiphoton microscopy at 2, 4, and 24 h post-application (Fig. 6C). In all images, the green fluorescence corresponds to the intrinsic autofluorescence of CUR, enabling direct visualization of drug distribution within the tissue without additional staining. Quantitative analysis of fluorescence depth using Fiji software provided objective measurements of penetration profiles over time. CUR-NCs exhibited a penetration depth of approximately 50 µm at both 2 h and 4 h, which further increased to 60 µm at 24 h, indicating sustained diffusion into the mucosal layers. In contrast, CS–CUR-NCs showed lower penetration depths (30 µm at 2 h, 35 µm at 4 h, and 40 µm at 24 h). Although CS is well-known for its mucoadhesive properties, the fluorescence micrographs revealed that its presence promoted a more extensive and intense fluorescent layer at the tissue surface, suggesting stronger retention but limited vertical diffusion. This behaviour supports the hypothesis that CS enhances surface adhesion, potentially slowing deep penetration while favouring prolonged residence at the epithelial interface. Remarkably, HA–CS–CUR-NCs demonstrated the highest penetration capacity, reaching 60 µm at both 2 h and 4 h and increasing to 72 µm at 24 h. The three-dimensional reconstructions confirmed a more homogeneous fluorescence distribution across the tissue section, indicating that this formulation successfully integrates multiple mechanisms: (i) the intrinsic penetration capability of CUR-NCs, (ii) improved surface retention provided by CS and (iii) enhanced interaction with the mucosal matrix mediated by HA. The synergistic combination of these components appears to promote both lateral distribution and deeper tissue permeation, overcoming the limitations observed with single-polymer systems. Finally, the PM containing all formulation components exhibited minimal penetration and a markedly weak, heterogeneous fluorescence signal, confirming that simple co-administration of the raw materials does not reproduce the performance of the nanostructured system. Collectively, these findings demonstrate that rational NC surface engineering significantly enhances mucosal penetration and promotes homogeneous drug distribution within the tissue, highlighting the potential of HA–CS–CUR-NCs as a robust platform for effective mucosal delivery of hydrophobic compounds.

Finally, the present work advances the field by replacing weak adsorption with covalent grafting of HA onto a CS-coated CUR-NCs core. This “graft-to” strategy locks the HA chains in place, conferring long-lived steric repulsion that is resistant to dilution or competitive displacement. More importantly, HA provides a bioresponsive motif; owing to its reported affinity for CD44 receptors,^57^ it may facilitate receptor-mediated interactions with mucosal and epithelial cells, while its hydrophilicity and negative charge create a hydration shell that minimises muco-adhesion and facilitates deep diffusion.^58^ Compared to conventional PEGylated or poloxamer-coated systems, the HA–CS conjugate offers dual functionality, improved colloidal stability and selective tissue engagement.^59^ These effects, together with the observed increases in mucosal deposition and cellular uptake, underscore the importance of fine-tuned surface properties in enhancing drug delivery performance. Moreover, considering that macrophages can actively internalise NCs and impact mucopenetration, the HA–CS–CUR-NCs system appears to at least partially overcome this clearance mechanism. These insights pave the way for the translational advancement of HA-functionalised NCs as next-generation drug delivery systems, particularly in applications where mucosal, transdermal, or transmucosal penetration is required.

## 4. Conclusion

This work underscores the potential of HA-functionalised CS-coated NCs as a versatile and rationally engineered platform for mucosal drug delivery. Beyond achieving covalent HA grafting and demonstrating improved *ex vivo* mucopenetration, the system embodies a strategic convergence of NC-based drug solubilization with stable, biointeractive surface design. The versatility of this approach opens the door to its application with a wide range of poorly soluble drugs and therapeutic molecules, particularly where mucosal targeting and barrier traversal are critical. Looking ahead, *in vivo* validation of pharmacokinetics, biodistribution, and therapeutic performance will be key to assessing clinical translatability. Altogether, the findings presented here offer a promising step toward next-generation nanomedicine platforms that not only overcome physicochemical formulation hurdles but also engage biological systems with precision and purpose.

## Author contributions

M. G. F.: conceptualization, methodology, validation, formal analysis, investigation, data curation, writing – original draft, writing – review & editing, and visualization. O. E. F.: conceptualization, methodology, validation, formal analysis, investigation, data curation, writing – review & editing, and visualization. A. R.: conceptualization, methodology, validation, formal analysis, investigation, and data curation. J. A. C.: resources and supervision. A. J. P.: conceptualization, resources, writing – review & editing, supervision, and project administration.

## Conflicts of interest

There are no conflicts to declare.

## Acronyms

ATR-FTIR: Attenuated total reflectance–Fourier transform infrared spectroscopy
CS: Chitosan
CS–CUR-NCs: Chitosan-curcumin nanocrystals
CUR: Curcumin
CUR-NCs: Curcumin nanocrystals
DLS: Dynamic light scattering
DMEM: Dulbecco's modified Eagle medium
DSC: Differential scanning calorimetry
EDC: 1-Ethyl-3-(3-dimethylaminopropyl)carbodiimide
H′-NMR: Proton nuclear magnetic resonance
HA: Hyaluronic acid
HA–CS–CUR-NCs: Hyaluronic acid–chitosan–curcumin nanocrystals
HPLC: High-performance liquid chromatography
MPM: multiphoton microscope
NCs: Nanocrystals
NHS: *N*-Hydroxysuccinimide
POL 188: Poloxamer 188
POL 407: Poloxamer 407
PBS: Phosphate buffered saline
PDI: Polydispersity index
PM: Physical mixture
RPMI: Roswell park memorial institute medium
SEM: Scanning electron microscopy
TEM: Transmission electron microscopy
TGA: Thermogravimetric analysis

## Data Availability

The datasets generated and analysed during the current study are available from the corresponding author upon reasonable request.
